# Development of an ultra‐thin parallel plate ionization chamber for dosimetry in FLASH radiotherapy

**DOI:** 10.1002/mp.15668

**Published:** 2022-04-22

**Authors:** Faustino Gómez, Diego M. Gonzalez‐Castaño, Nicolás Gómez Fernández, Juan Pardo‐Montero, Andreas Schüller, Alessia Gasparini, Verdi Vanreusel, Dirk Verellen, Giuseppe Felici, Rafael Kranzer, Jose Paz‐Martín

**Affiliations:** ^1^ Department of Particle Physics University of Santiago Santiago de Compostela Spain; ^2^ Radiation Physics Laboratory University of Santiago Santiago de Compostela Spain; ^3^ Group of Medical Physics and Biomathematics Instituto de Investigación Sanitaria de Santiago (IDIS) Santiago de Compostela Spain; ^4^ Department of Medical Physics Complexo Hospitalario Universitario de Santiago de Compostela Santiago Spain; ^5^ Physikalisch‐Technische Bundesanstalt (PTB) Braunschweig Germany; ^6^ Department of Radiotherapy Iridium Network Belgium; ^7^ Faculty of Medicine and Health Sciences University of Antwerp Antwerp Belgium; ^8^ SCK CEN Research in Dosimetric Applications Mol Belgium; ^9^ SIT Aprilia Italy; ^10^ PTW Freiburg Germany; ^11^ University Clinic for Medical Radiation Physics Medical Campus Pius Hospital Carl von Ossietzky University Oldenburg Germany

**Keywords:** FLASH radiotherapy, ionization chamber, ultra‐high dose rate

## Abstract

**Background:**

Conventional air ionization chambers (ICs) exhibit ion recombination correction factors that deviate substantially from unity when irradiated with dose per pulse magnitudes higher than those used in conventional radiotherapy. This fact makes these devices unsuitable for the dosimetric characterization of beams in ultra‐high dose per pulse as used for FLASH radiotherapy.

**Purpose:**

We present the design, development, and characterization of an ultra‐thin parallel plate IC that can be used in ultra‐high dose rate (UHDR) deliveries with minimal recombination.

**Methods:**

The charge collection efficiency (CCE) of parallel plate ICs was modeled through a numerical solution of the coupled differential equations governing the transport of charged carriers produced by ionizing radiation. It was used to find out the optimal parameters for the purpose of designing an IC capable of exhibiting a linear response with dose (deviation less than 1%) up to 10 Gy per pulse at 4 μs pulse duration. As a proof of concept, two vented parallel plate IC prototypes have been built and tested in different ultra‐high pulse dose rate electron beams.

**Results:**

It has been found that by reducing the distance between electrodes to a value of 0.25 mm it is possible to extend the dose rate operating range of parallel plate ICs to ultra‐high dose per pulse range, at standard voltage of clinical grade electrometers, well into several Gy per pulse. The two IC prototypes exhibit behavior as predicted by the numerical simulation. One of the so‐called *ultra‐thin* parallel plate ionization chamber (UTIC) prototypes was able to measure up to 10 Gy per pulse, 4 μs pulse duration, operated at 300 V with no significant deviation from linearity within the uncertainties (ElectronFlash Linac, SIT). The other prototype was tested up to 5.4 Gy per pulse, 2.5 μs pulse duration, operated at 250 V with CCE higher than 98.6% (Metrological Electron Accelerator Facility, MELAF at Physikalisch‐Technische Bundesanstalt, PTB).

**Conclusions:**

This work demonstrates the ability to extend the dose rate operating range of ICs to ultra‐high dose per pulse range by reducing the spacing between electrodes. The results show that UTICs are suitable for measurement in UHDR electron beams.

## 1 INTRODUCTION

Radiotherapy with ultra‐high dose rate (UHDR), exploiting the so‐called FLASH effect, has been recently proposed as a new treatment delivery technique with promising results at preclinical level.^1^, ^2^, ^3^, ^4^ This UHDR regime employs beams with mean dose rate in excess of 40 Gy/s.^1^, ^5^ The current available active detectors developed for the verification of conventional radiotherapy beams presents several problems when operating in UHDR regimes.^6^ It is therefore necessary to develop and conceptualize new active devices that allow accurate dosimetric measurements under such extreme dose delivery conditions.

Of particular concern is the case of vented air ionization chambers (ICs). These devices are used in radiotherapy departments for both relative and absolute dosimetry, and at calibration facilities as a secondary standard for the measurement of absorbed dose to water. However, their usage in UHDR beams is compromised by high charge recombination losses, leading to large correction factors exceeding 1.7 or more.^7^ This effect is a combination of the electric field perturbation inside the chamber together with the overlapping of large charge carrier densities produced by the beam delivery. Enhancing the charge collected due to free electrons, with higher mobility, is a way to reduce the recombination losses. In addition, the analytical theories so far employed to describe and correct the general recombination effects^8^, ^9^, ^10^, ^11^ fail in the UHDR regime, compromising the use of ICs for future clinical application in this type of delivery.

As a result it is important to develop detectors that are reliable for the dosimetric characterization of UHDR treatments to facilitate the implementation of this methodology in the clinic.^12^ One of the more straightforward approaches to improve the IC performance is the reduction of the electrode distance.^12^, ^13^ This work presents experimental results obtained with two *ultra‐thin* parallel plate ionization chamber (UTIC) prototypes with 0.27 and 0.22 mm gap, designed for reducing losses due to ion recombination in UHDR beams. These prototypes were tested in two different facilities, namely: PTB (Braunschweig) and SIT (Aprilia). The term “ultra‐thin” is used here as an expression of the strategy taken in this research work to reach the limits of operation of a parallel plate IC by reducing the electrode distance to the minimum value achievable. Optimal design parameters were obtained from an ion recombination model developed for this scope. Results show that UTIC can be used in UHDR dosimetry with ion recombination correction factors close to unity.

## 2 MATERIAL AND METHODS

### 2.1 Modeling recombination in an IC at UHDR

In the UHDR regime, Boag‐like analytical correction factors for ion recombination effects do not reproduce accurately the response of an IC at UHDR.^14^ For this reason, a detailed simulation of a parallel plate IC response has been carried out to find the optimal design parameters to obtain a linear dose response varying dose per pulse up to ultra‐high values. In this model, the coupled partial differential equations describing the one‐dimensional charged carrier transport inside an IC have been solved numerically. This approach has been explored in the past with satisfactory results for liquid ICs^15^, ^16^ and air ICs at intermediate dose per pulse range.^13^, ^17^ For the particular case of a parallel plate IC, the following set of equations are used to describe the charge transport in the sensitive air volume in the IC, being *x* the coordinate perpendicular to the electrodes and *t* the time:

(1) $$\begin{array}{ccc} \frac{\partial n_{+}\left(x,t\right)}{\partial t} & = & I\left(x,t\right)-\alpha \;n_{+}\left(x,t\right)\;n_{-}\left(x,t\right)\\ & & -\;\frac{\partial }{\partial x}\left[E\left(x,t\right)\;\mu_{+}\;n_{+}\left(x,t\right)\right]\\ \frac{\partial n_{-}\left(x,t\right)}{\partial t} & = & \gamma \;n_{e}\left(x,t\right)-\alpha \;n_{+}\left(x,t\right)\;n_{-}\left(x,t\right)\\ & & +\;\frac{\partial }{\partial x}\left[E\left(x,t\right)\;\mu_{-}\;n_{-}\left(x,t\right)\right]\\ \frac{\partial n_{e}\left(x,t\right)}{\partial t} & = & I\left(x,t\right)-\gamma \;n_{e}\left(x,t\right)+\frac{\partial }{\partial x}\left[v_{e}\left(x,t\right)\;n_{e}\left(x,t\right)\right], \end{array}$$

where $n_{i}$( i=+,−,e) are the positive ion, negative ion, and electron volumetric densities in m ^−3^. We denote E(x,t)as the electric field across the chamber. The source term I(x,t)represents the amount of charge carriers of either sign escaping initial recombination produced in the sensitive medium per unit volume and time in m ^−3^ s ^−1^. The parameter α in m ^3^ s ^−1^ accounts for the volume recombination effect between positive and negative ions. The attachment constant γ (inverse of the attachment lifetime τ), has been taken as a function of the electric field. Finally, $v_{e}$is the electron velocity and $\mu_{+}$and $\mu_{-}$denote the mobility of positive and negative ions, respectively. The electric field dependence across the chamber can be computed solving the one‐dimensional Poissonequation,

(2) $$\frac{\partial E(x,t)}{\partial x}=\frac{e}{\varepsilon }\left[n_{+}\left(x,t\right)-n_{-}\left(x,t\right)-n_{e}\left(x,t\right)\right]$$

being ε the dielectric constant of the medium. The bias voltage *V* provided to the chamber sets an additional boundary condition for the solution of the electric field. Considering that the electrodes are separated by a distance *d*, we assume that

(3) $$\int_{0}^{d}E\left(x,t\right)\;dx=V.$$

The above set of equations represents an approximation to the problem of describing air ICs in which effective parameters α, τ and average ion mobilities reduce the complexity of up to 200 individual molecular phenomena.^18^ For this work, the average ion mobilities in air were taken from the work of Zhang et al,^19^ the electronic velocity was simulated with the Magboltz code,^20^ and the electron attachment coefficient was taken from work by Boissonnat.^21^ Direct electron–ion recombination has not been included here since its contribution to the charge collection efficiency (CCE) has been estimated to be typically smaller than 0.1% in the cases considered in this work. The free electron fraction (FEF) is calculated as the fraction of electrons released by ionization in the chamber volume that reach the positive electrode.

For the numerical solution of the system of equations, a temporal and spatial discretization of Euler type were used. The discretization parameters were optimized to obtain a good compromise between accuracy and computational time. In the simulations, the distance between electrodes was discretized in 400 intervals with a time step during the radiation pulse delivery below 1.3 $\times 10^{-11}$s. The error in the CCE associated with this discretization was estimated to be below 0.1%.

### 2.2 Design of the ultra‐thin ionization chamber

**FIGURE 1:**
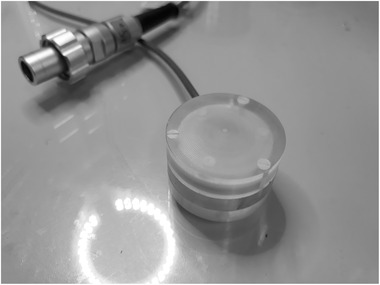
UTIC1 prototype with a distance between electrodes of 0.27 mm

**FIGURE 2:**
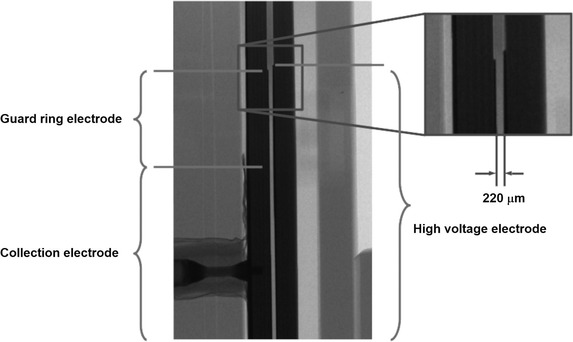
MicroCT transverse image of UTIC2 prototype. The distance between the electrodes of this prototype is 220 μm

The UTIC prototypes electrodes are built on two 30 mm radius and 1 mm thick FR4 disks. Electrodes are made of Cu‐Ni‐Au 18 μm thick layers. High voltage and guard ring electrodes have 10 mm external radius while the collection electrode radius is 5 mm. Between the guard ring and the collection electrode a 0.25 mm wide clearance is provided. Distance between electrodes is achieved by a laser‐machined 0.25 mm thick Mylar spacer that includes a lateral slit for air ventilation. Rexolite^®^ was used for the housing of the IC. The connection of the IC to the electrometer was provided by a triaxial cable with PTW‐M connector (Figure 1). Although the intended nominal distance between the electrodes was 0.25 mm, chamber geometry was verified after final assembly through an X‐ray image of the active volume (Figure 2) performed with a MicroCT (Bruker Skyscan 1272). It was found that the actual electrode distances were 0.27 and 0.22 mm for prototypes UTIC1 and UTIC2, respectively.

Since this work is focused on reducing recombination effect on IC, nontissue equivalent materials such as FR4 and Au‐Ni‐Cu were used in order to keep manufacturing process simple.

Two prototypes (UTIC1, UTIC2) were built for experimental characterization in order to validate the numerical results. One was tested in PTB (Physikalisch‐Technische Bundesanstalt, Germany) and the other at the ElectronFlash accelerator at SIT S.p.A (Aprilia, Italy).

### 2.3 Experimental measurements

**FIGURE 3:**
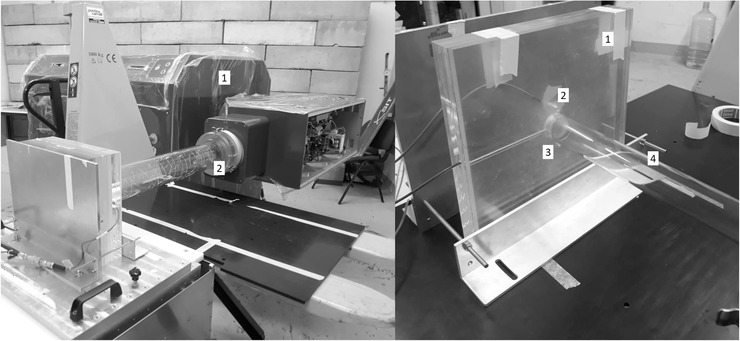
Experimental setup at SIT (Italy). The image on the left shows the ElectronFlash (linear accelerator) (1) using the 100 mm diameter applicator (2). On the right is the PMMA phantom (1) with the flash‐diamond (2) and the UTIC (3) prepared to be irradiated with the 35‐mm diameter applicator (4)

Measurements at SIT facility were performed using ElectronFlash accelerator, a research dedicated linac ( https://www.soiort.com/flash‐rt‐technology/ ). Such system operates in electron mode only, has two energies, 7 and 9 MeV; beam is collimated by poly methyl methacrylate (PMMA) applicators into fields from 1 cm diameter up to 12 cm diameter. Flatness and symmetry for the 10 cm field are better than 3%. Maximum dose rate achievable with applicator 10 cm is beyond 1000 Gy/s, with a dose per pulse above 4 Gy and the pulse repetition frequency of 250 Hz. Dose rate can be varied in a very wide range (0.03–10 000 Gy/s) by varying the time duration of the pulse, pulse repetition frequency (1–250 Hz at maximum peak power, otherwise up to 400 Hz) and applicator diameter. The dose per pulse was varied from approximately 10 to 1 Gy by changing the pulse duration from 4 to 0.1 μs. Measurements were performed using 50 pulses of the beam at 10 Hz repetition rate. A PMMA phantom was built for the insertion of the UTIC and a specially modified diamond detector (flash‐diamond) for UHDR^22^ which was used as a reference dosimeter (Figure 3). Flash‐diamond was placed at 30 mm transversal distance from UTIC axis at the same depth of material. A PMMA build‐up was added to achieve a water equivalent depth of 16.6 mm. The flash‐diamond used along with the UTIC has been recently developed and presents a linear response in this range of dose per pulse.^22^ It should be noted that for the 35 mm applicator the flash‐diamond served as reference field detector, placed outside of the beam, with a lower dose readout proportional to the dose delivered to the UTIC. Additional measurements at 4 μs pulse duration were performed placing the diamond in the reference point of measurement of the chamber inside the PMMA phantom to obtain a calibration of the dose per pulse. The UTIC was operated at 300 V and also at a reduced voltage of 150 V intentionally to show explicitly the increased ion recombination in a reduced electric field strength.

**FIGURE 4:**
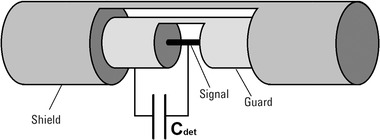
Scheme of capacitor connection $C_{det}$ of 100 nF in the triaxial cable for use of a standard electrometer in UHDR pulsed beam

All measurements at SIT were performed using a PTW UNIDOS E electrometer. For the dose per pulse we used, instantaneous current (i.e., current due to the 4 μs pulse duration) produced by the radiation pulse in the UTIC reach few mA. This considerably exceeds the maximum instantaneous electrical current specification of the electrometer. To overcome this problem, a 100 nF capacitor was added in parallel to the signal cable between the chamber and electrometer. This capacitor was connected between the core wire (collection electrode) and the inner shield of the triaxial cable (guard ring electrode) in order to reduce the maximal voltage at the electrometer input (Figure 4).

**FIGURE 5:**
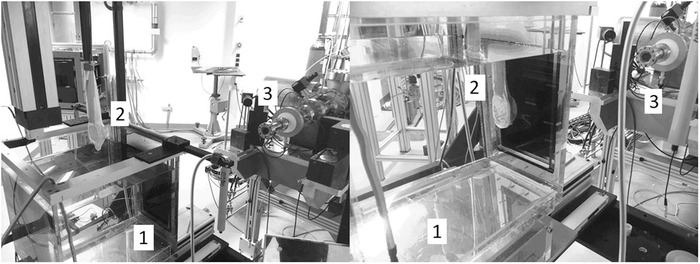
Experimental setup at PTB (Germany). Both images show the water tank (1), the UTIC (2) in a waterproof bag mounted on a xyz positioning system outside water (left) and in the water at reference depth (right) and the beam exit window of the beamline of the electron linear accelerator (3)

Measurements at PTB were performed in PTB's ultra‐high pulse dose rate reference electron beam,^23^ in a water tank varying the dose per pulse between low and high range with a fixed pulse duration of 2.5 μs at a nominal energy of 20 MeV and 5 Hz pulse frequency (Figure 5). The delivered dose per pulse is varied by modifying the width of a slit diaphragm (0.5–8.0 mm) at the beginning of the beamline, without influencing the position of the beam, determining the transmitted charge per beam pulse. Bias voltage used for the chamber was +250 V. The absorbed dose to water at UTIC effective depth of measurement was calibrated by means of PTB's alanine dosimetry system.^24^ Due to the fast ion collection time of the UTIC, below 3 μs at 300 V, there is no pulse overlapping in this chamber for standard linac deliveries and thus the pulse repetition rate is irrelevant when investigating the detector properties.

## 3 RESULTS AND DISCUSSION

### 3.1 Simulations for the IC design

**FIGURE 6:**
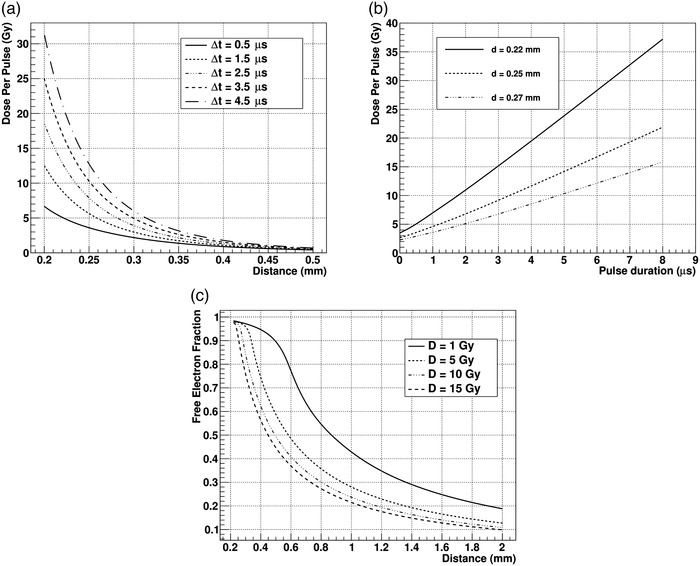
Simulation of the performance of an ionization chamber in UHDR. Panel (a) shows the curves of dose per pulse versus the distance between electrodes at which the chamber response deviation from linearity is 1% (charge collection efficiency of 99%) for different pulse duration from 0.5 μs up to 4.5 μs (assuming constant dose rate during the delivery). Panel (b) shows the relationship between dose per pulse and pulse duration for distances 0.22, 0.25, and 0.27 mm between electrodes at which the chamber response deviation from linearity is 1% (charge collection efficiency of 99%). Panel (c) shows the free electron fraction versus distance between electrodes for different dose per pulse delivered in a 4.5 μs pulse. All the simulated results presented here are calculated at 300 V bias voltage and standard temperature, pressure, and relative humidity conditions (20 ° C, 1013.25 hPa, and 50% respectively)

Based on the numerical solution of the system of coupled partial differential equations in Section 2, a detailed study of the design parameters that have a significant impact on the charge collection process was carried out. It was concluded, based on the results, that the distance between electrodes is by far the variable that has the greatest impact on improving the collection efficiency. The reason for this can be summarized in three main factors:

- Considering the usual operating voltage of an air IC (300–400 V), reducing the distance between electrodes increases the nominal electric field inside the chamber and, therefore, the carriers move faster, decreasing the probability of recombination.
- Reducing the distance between electrodes leads to a shorter path for the carriers to travel and, therefore, the charged carrier densities overlap for a shorter time.
- In an IC, as the distance between electrodes decreases, the FEF increases because the attachment probability is smaller following the reduction of electron drift time. The increase in the FEF can be clearly visualized in Figure 6c. Due to the short distance to the positive electrode, an UTIC detector pumps out negative charge (as free electrons) before it gets attached to oxygen species. The negative ion density in the chamber is actually reduced due to the negative charge already collected as free electrons. This prevents the recombination of that negative charge that in a bigger gap IC would be eventually attached to large molecules with much smaller mobility. This fast collection of the negative charge has also the undesired effect to produce a net positive charge accumulation close to the negative electrode of the chamber. As a consequence, even for ultra‐thin ionization chamber in high dose per pulse, the electric field perturbation is not negligible.

Using simulation, it is possible to predict the CCE of the chamber together with different parameters of operation like FEF, perturbation of the electric field, or ion collection time. Considering that the distance between electrodes is the key factor to reduce volume recombination, we have analyzed the dose per pulse at which CCE would yield 99% as a function of electrode distance, between 0.5 and 0.2 mm, and delivery duration. In this work, dose rate has always been considered constant during pulse delivery. Results are shown in Figure 6a. Respect to the volume recombination coefficient, we have analyzed different data sets of these prototype chambers using continuous and pulsed beams together with the results from SIT and PTB, obtaining that the highest likelihood value of α was $0.42\times 10^{-12}$m ^3^ s ^−1^. For these chambers with submillimetric electrode distance, a clear dependence of the operation limits on the instantaneous dose rate exists when the pulse duration is in the μs range. It is common, in the use of standard ICs, to assume that pulse duration is much shorter than charge collection time. For this reason, it is usually assumed that recombination correction only depends on dose per pulse. In UTIC under UHDR, this principle does not hold since ion collection time (less than 3 μs for 0.25 mm electrode distance operated at 300 V) can be of the same order as pulse duration. Therefore, dose per pulse has to be considered together with pulse duration (as shown in Figure 6a,b) or instantaneous dose rate. In Figure 6, the magnitude dose to air is indicated by default.

Setting the electrode separation to 0.22, 0.25, and 0.27 mm, we have estimated the dose per pulse that would produce a 99% CCE in the parallel plate chamber when using pulse duration up to 8 μs. This is shown in Figure 6b. It is important to appreciate the dramatic dependence of chamber performance on small gap variations. This represents a challenging technical issue in the assembly and operation of this type of chambers.

Based on simulation results, it can be concluded that an IC with a gap around 0.25 mm would be suitable for UHDR measurements with ion recombination losses below 1% for deliveries with dose per pulse less than 12 Gy and pulse duration above 4.5 μs using 300 V bias voltage.

**FIGURE 7:**
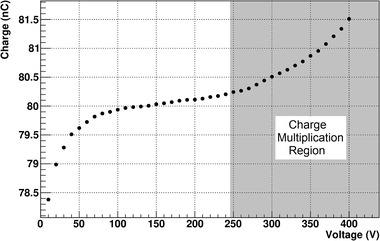
Charge versus voltage curve for a 0.22 mm UTIC irradiated with a continuous beam of 50 kV X‐ray and 1.5 mA current without added filtration and estimated dose rate of 4 Gy/s

It must be taken into account that the technique of reducing the IC gap has certain intrinsic physical limitations. First of all, the electrode distance, as indicated before, is a critical parameter that has to be extremely constant. Electric field increase can lead to deformation of electrodes, which must be sufficiently robust to be able to overcome the electromagnetic force. Second, the technological capacity to manufacture a chamber with this small gap is challenging since variations of tens of micrometers can lead to measurable differences of behavior in relation to recombination effects. Linked to this, thermal expansion and contraction of the chamber materials could cause a significant change in the gap, again with measurable effect in the performance. Finally, due to the large electric field used for chamber bias, charge multiplication may also be triggered. To illustrate the charge multiplication effect, Figure 7 shows the charge collected in a 0.22‐mm gap prototype as a function of the chamber voltage irradiated with a continuous beam of 50 kV X‐rays and 1.5 mA current with no added filtration. From this chamber measurements, we can clearly observe the exponential increase of collected charge due to electron multiplication at high electric fields. Although it is possible to measure in a regime with charge multiplication,^25^ it is preferable to avoid the existence of charge currents that do not have an intrinsic dosimetric origin. Also, if the gap is small enough (or the electric field large enough), undesirable discharges can occur inside the chamber. These physical factors set a limit on the minimum electrode distance and maximum operating voltage that can be used in these type of chambers.

In the existing literature, the FEF (inherited from Boag's formalism) appears as a constant that depends only on the electric field and distance between electrodes. Experimental data and simulation indicate that this constancy of the FEF does not hold for large dose per pulse deliveries. Consequently, for example, all the efforts to reformulate the models in the UHDR keeping that parameter as a constant are probably starting from a wrong assumption. In addition, the detailed description of air ICs under UHDR is complex due to the multiplicity of physical interaction processes present in the air and the high nonlinearity of the charge carrier distributions involved. Even using a numerical approach, like the one considered in this work, many of the parameters involved (i.e., volume recombination coefficients, electron attachment lifetime, or average ion mobilities) have still a large uncertainty. For that reason, additional research effort is needed to elaborate a more accurate and reliable description of this ionometric standards under UHDR.

### 3.2 Experimental measurements

**FIGURE 8:**
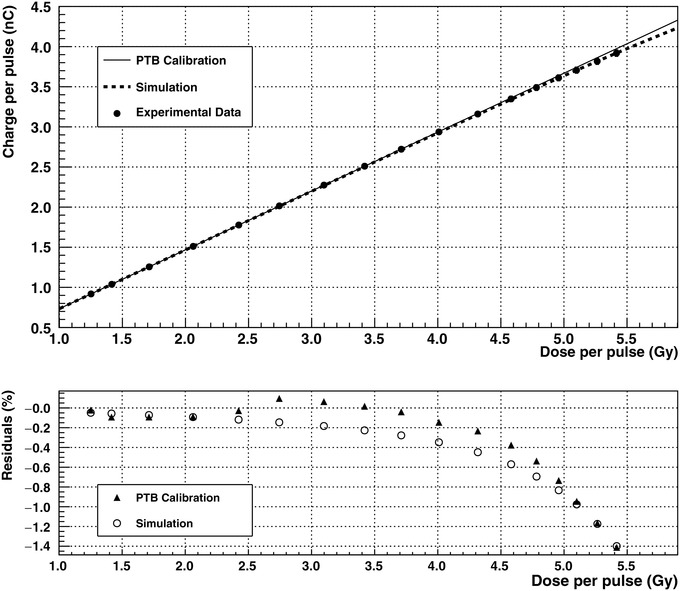
Results obtained at PTB in the water tank for the UTIC1 prototype at +250 V bias voltage. The experimental charge obtained versus dose per pulse is indicated by filled circles in the upper graph. The sensitivity obtained for this chamber was used to extrapolate the ideal linear response at high dose per pulse indicated by continuous line. Dashed line corresponds to expected results from simulation. Residuals expressed in terms of local relative differences are shown below were filled triangles correspond to experimental data and circles to simulation. For simulation, the volume recombination constant α was fixed to 0.42 $\times 10^{-12}$m ^3^ s ^−1^

Measurements obtained with prototype UTIC1 at PTB are shown in Figure 8, indicating the linearity of the collected charge as a function of the dose per pulse delivered. Chamber sensitivity of 0.7337 nC/Gy obtained at low dose per pulse was used to extrapolate the expected chamber response in the UHDR regime and find recombination deviations. Figure 8 also shows the expected performance from the numerical simulation for the chamber nominal gap. There is a good agreement between data and simulation except in the low dose per pulse region where the experimental data showed some discrepancies (0.2–0.3%) with respect to the linear regression. In this electron beam, the recombination effect produces a reduction of the collected charge of 1.4% at 5.4 Gy per pulse. Simulation data suggest that, increasing the chamber voltage to 300 V will lead to a reduction in the recombination losses from 1.4% to 0.3%.

**FIGURE 9:**
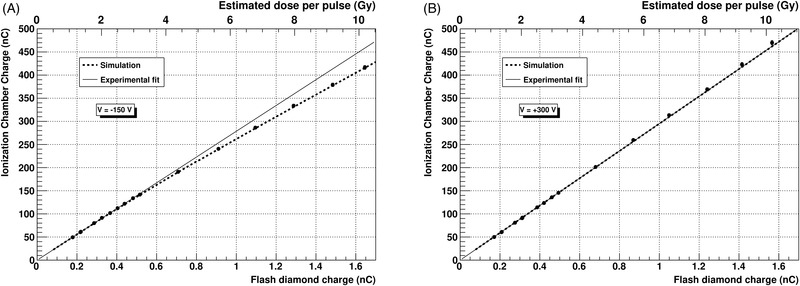
UTIC2 chamber response tested in the 9 MeV flash electron beam at SIT in a PMMA phantom. The UTIC2 absolute collected charge at +300 V (b) and −150 V (a) bias voltage is correlated with the flash‐diamond readout used as reference field detector. Solid line indicates the ideal linear chamber response while the dashed line corresponds to the expected performance from simulation. For simulation, the volume recombination constant α was fixed to 0.42 $\times 10^{-12}$m ^3^ s ^−1^

In Figure 9, we show the response with dose per pulse of the prototype UTIC2, tested at the ElectronFlash linac (SIT). In this case, a flash‐diamond was used as a reference field detector to verify the linearity of the IC with respect to the dose delivered. The UTIC2 prototype operated at +300 V bias voltage shows a linear response with dose per pulse up to 10 Gy (Figure 9b). Intentionally, the chamber was also operated at −150 V bias voltage to enhance the recombination effect and make it visible (Figure 9a). Measurements performed at SIT showed a polarity effect below 0.3% for this chamber.

## 4 CONCLUSIONS

We have performed a numerical simulation of parallel plate air IC's charge transport under ionizing radiation in order to study their behavior in UHDR beams. Reduction of the electrode distance in a parallel plate chamber appears as a key factor in order to extend the range of ICs into the region of ultra‐high dose per pulse. Simulations showed that to access the current requirements also in UHDR (i.e., 99% CCE for 5–10 Gy per pulse in 2.5 μs), it would be necessary to use a chamber with a gap lower than 0.30 mm. In addition, the simulated performance of these chambers indicate a dramatic dependence of the device with the actual electrode distance, where variations of tens of micrometers can produce measurable changes of performance. On the other hand, the enhancement of the electric field makes these chambers prone to charge multiplication if operated at fields close to 1200 V/mm.

Considering the challenging task of constructing such a small gap chamber, two prototypes were built with electrode distance of around 0.25 mm (0.22 and 0.27 mm). The performance of these two vented chamber prototypes (UTIC1, UTIC2) was tested at two UHDR electron beams, namely: PTB MELAF facility (Braunschweig) and electron‐flash linac at SIT (Aprilia). Measurements performed at PTB with UTIC1, with a polarizing voltage of +250 V, showed recombination losses of 1.4% at 5.4 Gy per pulse, for a pulse duration of 2.5 μs. In the tests at SIT with UTIC2, the dose per pulse was varied changing the pulse duration of a 9 MeV electron beam with a 35 mm diameter PMMA applicator. In this case, a negligible deviation with respect to linear response was measured when operating the at +300 V with a dose per pulse up to 10 Gy with 4 μs duration, when compared with a flash‐diamond response. The different experimental results are well reproduced by the numerical simulation approach. We have demonstrated that such UTIC, with ∼ 0.25 mm electrode distance, operated at 300 V, can work under UHDR beams with dose per pulse less than 12 Gy and pulse duration above 4.5 μs, having a CCE in excess of 99%. The availability of these ultra‐thin chambers can play a substantial role to allow the application of ICs for UHDR beams, providing a way to extend the current code of practice for clinical beam dosimetry based on ionometric standards.

## CONFLICT OF INTEREST

Rafael Kranzer is a PTW employee. Giuseppe Felici is an SIT employee.
